# Combining group psychotherapy and yoga exercises improves quality of life in mental health professionals: a controlled randomized clinical trial

**DOI:** 10.1108/MIJ-10-2019-0007

**Published:** 2019-11-04

**Authors:** Marilena Maglia, Roberta Auditore, Stefano Pipitone, Rachele DiPasqua, Lucio Inguscio, Pasquale Caponnetto

**Affiliations:** CTA-Villa Chiara Psychiatric Rehabilitation Clinic and Research, Mascalucia, Italy and University of Catania, Catania, Italy; CTA-Villa Chiara Psychiatric Rehabilitation Clinic and Research, Mascalucia, Italy; Vidya Vahini Trust, Uttarkashi, India and Yoga Vidya, Siracusa, Italy; CTA-Villa Chiara Psychiatric Rehabilitation Clinic and Research, Mascalucia, Italy; Department of Psychology, University of Rome La Sapienza, Rome, Italy; University of Catania, Catania, Italy

**Keywords:** Group psychotherapy, Psychological intervention

## Abstract

**Purpose:**

This study aims to investigate the effects of combining 12-week group psychotherapy with yoga exercises on stress perception and quality of life in mental health professionals.

**Design/methodology/approach:**

This study was a parallel-arm randomized controlled trial, in which the participants was unaware of which group was the experimental one. Participants involved in our research were assigned to two groups of separate treatment that followed for three months group psychotherapy combined with yoga program for stress management or usual stress coping strategies.

**Findings:**

The findings did not reveal a significant difference in stress perception assessed in the two groups either before or after intervention but reveal a significant difference in the quality of life in the two groups before and after the psycho behavioral interventions.

**Originality/value:**

The findings did not reveal a significant difference in stress perception assessed in the two groups either before or after intervention but reveal a significant difference in the quality of life in the two groups before and after the psycho behavioral interventions.

## 1. Introduction

Mental health professionals (psychiatrists, nurses, psychologists, social workers and occupational therapists) are often under time constraints experiencing stress during their interactions with patients who are potentially violent or at risk for suicide, often called upon to maintain a positive attitude and enthusiasm, despite high work demands. In these conditions, experts can feel distressed that affects their psychological well-being. Such stressful conditions might eventually lead to burnout. Moreover, stress indirectly affects organization and even patients themselves (Burnard, 1991).

The types of group psychotherapy interventions could be distinguished in psycho-education, cognitive behavioral oriented “stress management/coping skills training”, psychodynamic therapy, experiential existential centered therapy and mindfulness-based stress reduction training. Group psychotherapy outcome studies have demonstrated findings supporting the efficacy of this treatment format (Burlingame *et al.*, 2003). In addition to its established efficacy, group therapy has been shown to be a more cost effective format of treatment and managed care organizations are encouraging its use in outpatient settings (Taylor *et al.*, 2001). Several studies have demonstrated that group treatment is effective and outcome studies have provided evidence of group psychotherapy efficacy for the treatment of various psychological concerns (Burlingame *et al.*, 2003).

Yoga Vidya is a body–mind exercise, which promotes physical, mental and spiritual health. Yoga and Ayurvedic knowledge underline that the cause of diseases and stress is related to unbalanced *prana* (Bhavanani *et al.*, 2014).

Yoga practice may make individuals more capable of working better in highly stressful situations (Louie, 2014). Primarily, studies of yoga effects on psychological well-being in medical ﬁeld focus on nursing staff (Chang *et al.*, 2004; Chen *et al.*, 2013; Chung *et al.*, 2014; Gomes and Teixeira, 2014; Liu and Liu, 2009; Tang *et al.*, 2005). Few studies have measured effects of yoga on psychological well-being and stress adaptation in different mental health professionals (Chang, 2003; Shen *et al.*, 2005; Lin *et al.*, 2015).

To our knowledge, there are no studies in the world that have evaluated the effects on psychological well-being of combining group psychotherapy and yoga exercises for mental health professionals and in this sense this would be the first study in the world.

Therefore, we conducted a pilot study combining group psychotherapy and yoga exercises for mental health professionals). We hypothesized that combining group psychotherapy and yoga exercises could effectively improve psychological well-being by reducing stress perception and increasing the quality of life of mental health professionals by well designed and implemented group psychotherapy plus yoga classes. Based on this hypothesis, the principal purpose was to investigate the effects of 12-week group psychotherapy combined with yoga exercises intervention psychological well-being, stress perception and quality of life in mental health professionals to verify its acceptability and efficacy.

## 2. Research methods

### 2.1 Study design

This study was conducted at CTA-Villa Chiara Psychiatric Rehabilitation Clinic and Research, situated in Mascalucia (Catania, Italy). This study was a parallel-arm randomized controlled trial, in which the analyzer was unaware of which group was the experimental one. Participants involved in our research were assigned to two groups of separate treatment that followed for three months group psychotherapy combined with yoga program for stress management or usual stress coping strategies (stress counseling service).

The participants were randomly assigned to each group, and the EuroQol VAS and the perception stress scale (PSS), were evaluated and compared at *baseline* (T0) and after three months (T1).

The experimental condition (group psychotherapy combined with yoga) consisted of one weekly session of 2 h for a total duration of 12 weeks (1 h of group psychotherapy and 1 h of yoga exercises). The training was modeled on the patient, considering the baseline assessment.

Those who were assigned to the control group participated in a free tea-time during which they watched television and did not exercise. The participants signed informed consent prior to enrolling in the study.

An experienced clinical psychologist led the group and talked about stress management, problem-solving, coping and cognitive reframing to examine and deal with negative thoughts, from cognitive-behavioral theory (Petrocelli, 2002).

The sessions of Yoga Vidya exercise involved postures (asana, in Sanskrit), breathing exercises (pranayama, in Sanskrit), some concentration exercises (meditation) and relaxation (Saraswati, 2017).

### 2.2 Research participants

The research participants were 37 mental health professionals who worked in CTA-Villa Chiara Psychiatric Rehabilitation Clinic and Research and who were not involved in any formal exercise program. They were randomly assigned to the psychotherapy plus yoga group or control group. The inclusion criteria consisted of being mental health professionals who were not involved in a formal exercise program and who were willing to participate in this study. Exclusion criteria included pain due to injuries to shoulders, waist or lower back, and musculoskeletal diseases such as muscle strains that made participants unsuitable to participate. The participants were recruited because of poster advertizement and in person. After we had received registration, a meeting was held to explain the details of the trial. Then, the participants signed the informed consent form and after having checked that all the necessary criteria for inclusion (or exclusion) were satisfied, patients were first admitted to the study and subsequently assigned to the sexperimental group or to the control group, because of the computer-generated random number tables. Therefore, any statistically significant differences between the two groups at baseline were excluded. It was expected that the two groups were homogeneous by drawing lots of random allocation. After randomization, outcome variables were measured at the beginning (baseline) and at the end of the program (twelve weeks later).

### 2.3 Research measures

The tools of the study consisted of two subjective self-administered scales (EuroQol VAS and PSS). Before and after the group psychotherapy plus yoga exercise intervention, all participants were asked to complete two self-administered scales (EuroQol VAS and PSS), after having indicated demographic characteristics (e.g. gender, age, educational status and years of work) and professional background.

The EuroQOL five dimensions questionnaire (EQ-5D) is one of the most commonly used generic questionnaires to measure health-related quality of life (HRQOL). It consists of a questionnaire and an analog visual scale (EQ-VAS). The EQ-VAS is a self-rated health status using a VAS. The EQ-VAS records the subject’s perceptions of their own current overall health and can be used to monitor changes with time. The self-assessment questionnaire is a self-reported description of the subject’s current health in five dimensions i.e. mobility, self-care, usual activities, pain/discomfort and anxiety/depression. The subject is asked to grade their own current level of function in each dimension into one of three degrees of disability (severe, moderate or none). Each health state can be ranked and transformed into a single score called the utility. The utility score is an expression of the Quality Adjusted Life Years (QALY) and is commonly used to make evidence-based decisions in analyzes of cost-effectiveness. In addition, there is an analog visual scale (VAS) to indicate the general health status in which 100 indicates the best health status.

The PSS is the most widely used psychological instrument for measuring the perception of stress. It is a measure of the degree to which situations are appraised as stressful in one’s life. Items were designed to assess how unpredictable, uncontrollable and overloaded respondents find their lives to be. The scale also includes many direct queries about current levels of experienced stress. Moreover, the questions are general, hence they are relatively free of content specific to any sub-population group. The questions in the PSS ask about feelings and thoughts during the past month. In each case, respondents are asked how often they felt a certain way (Sheldon Cowen). The PSS comprises 14 items with a five-point scale, ranging from Never (0) to Very often (4) and higher scores (maximum score = 56), which represent higher levels of perceived stress.

## 3. Results

### 3.1 Flow of participants

The 31 individuals who completed this research were 19 men and 12 women, in total (a yoga Vidya intervention group and a control group).

Tables I and II show the socio-demographic characteristics of the two groups of participants.

**Figure 1:**
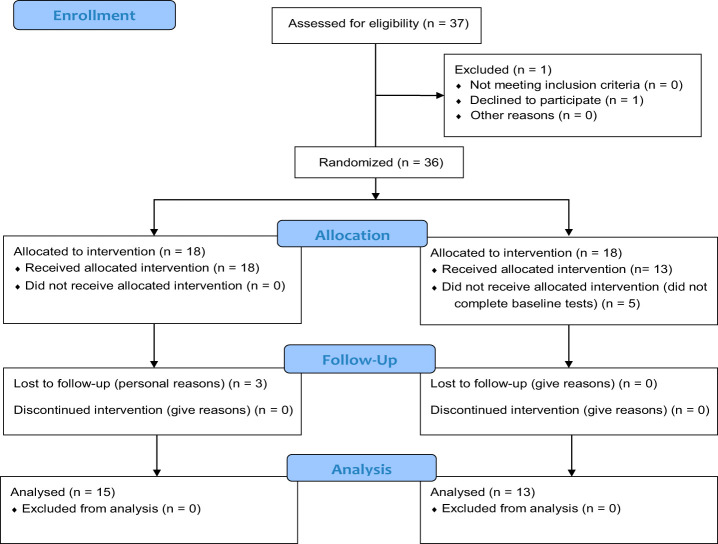
CONSORT 2010 flow diagram

In total, 37 individuals (*n* = 37) were deemed eligible; one withdrew prior to baseline because of personal reasons. In total, 36 individuals provided written informed consent and were randomized to the yoga group (18) or control group (18). During allocation, five participants in the control group did not complete baseline testing. In the psychotherapy plus yoga group, three participants failed to complete follow-up testing Figure 1.

### 3.2 Statistical analysis

For the statistical analysis of the data and for their manipulation, the software Microsoft Excel was used, while the IMB software SPSS 24 was used for statistical analysis, with the objective of assessing over time variations of the results obtained through the administration of the rating scales used at baseline (T0) and after 3 months (T1). To assess the impact of the group psychotherapy plus yoga exercises treatment an analysis of variance (ANOVA), mixed with repeated measures, was performed, with the two types of group applied as factors between subjects and the two temporal observations (T0 and T1) as factors within subject, also by analyzing the “interaction time X treatment”.

As the first step, we analyzed whether group condition (psychotherapy plus yoga group and control group) and stress level categories (below average, average, high or medium, high) are independent of one another.

**Figure 2:**
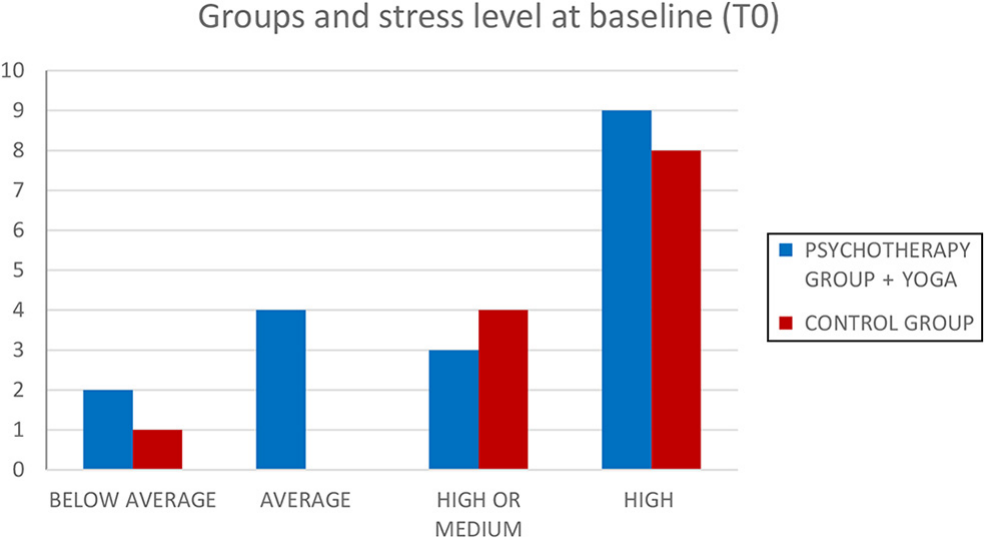
Number of Ss for each group condition across different stress level categories at baseline (T0)

We analyzed the entire sample using a contingency table at baseline (T0) and after three months (T1). Figure 2 shows the frequencies for each group condition across different stress level categories at baseline (T0).

The chi-square (*χ*^2^) statistic is 1.8641. The *p*-value is 0.601078. The result is not significant at *p* < 0.05.

**Figure 3:**
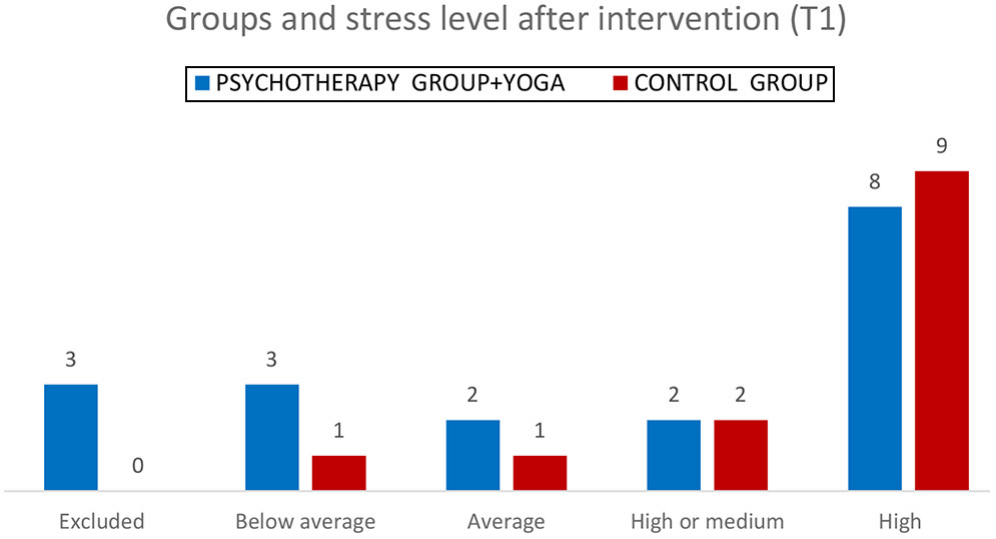
Number of Ss for each group condition across different stress level categories after intervention (T1)

Figure 3 shows the frequencies for each group condition across different stress level categories after intervention (T1).

The *χ^2^* statistic is 1.2557. The *p*-value is 0.739677. The result is not significant at *p* < 0.05.

Having analyzed the entire sample (intervention, non-intervention) at baseline (T0) and examining individually the mean of the results obtained in the test batteries (EuroQol VAS and PSS) used in the assessment step at baseline, we found that after three months (T1), there was a significant difference in Qol-VAS scores between the group psychotherapy group combined with yoga exercises group and the control group F (1,26) = 11.15 and *p* = 0.003.

**Figure 4:**
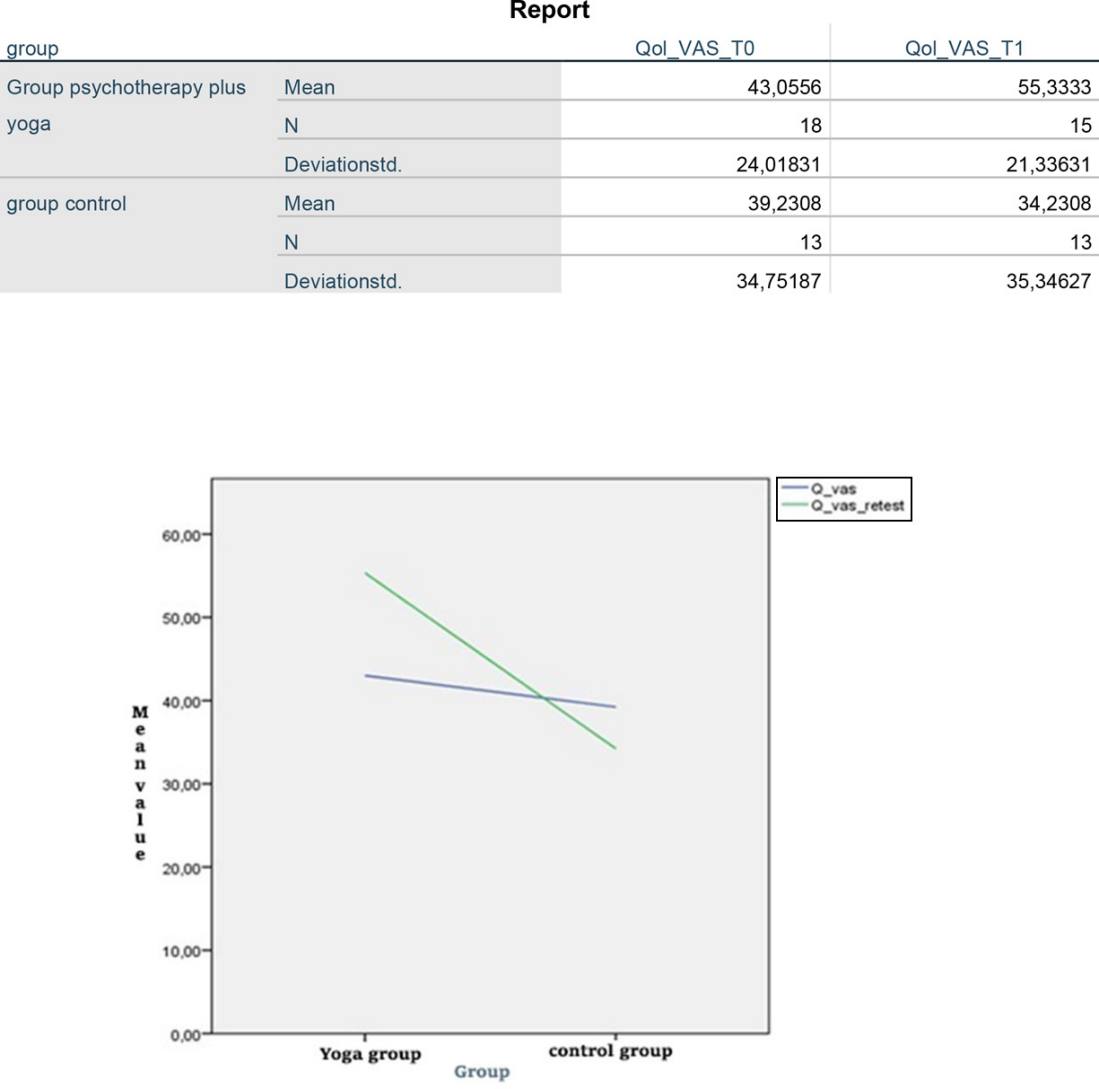
Report

**Figure 5:**
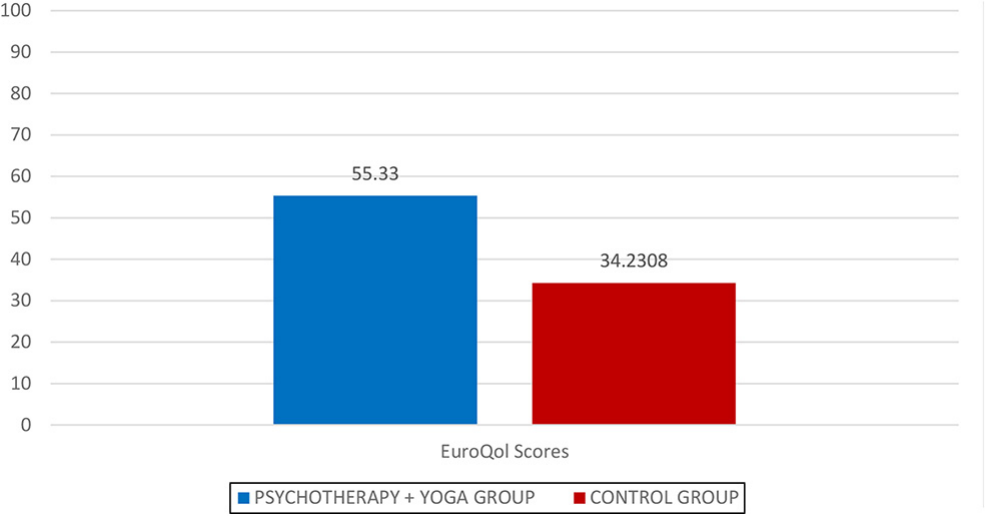
Euro-QoL scores recorded after intervention (T1)

The median QoL-VAS score before in the group that received group psychotherapy group combined with yoga intervention was 43.0556, which it was increased to 55.3333 after the intervention. The statistical analysis revealed a significant difference in the quality of life assessed by Euro-QoL VAS between group psychotherapy group combined with yoga vs control group Figures 4 and 5.

**Figure 6:**
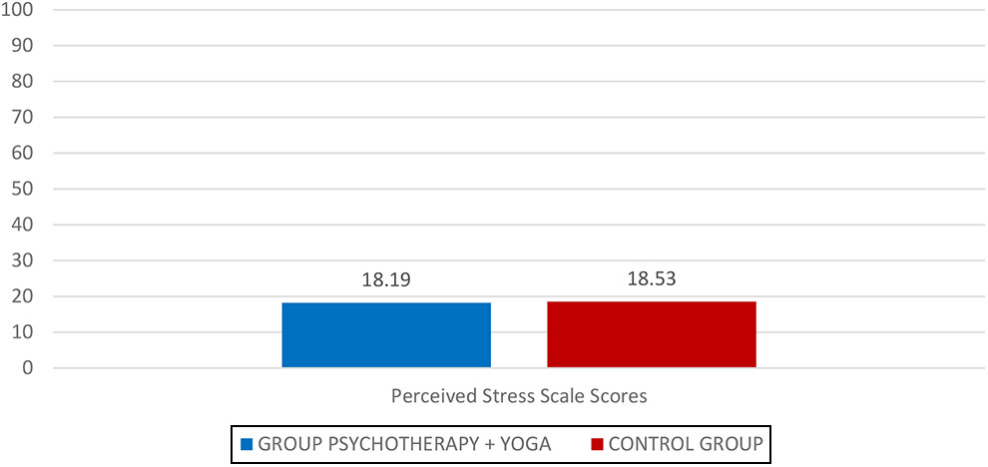
PSS scores recorded after intervention (T1)

The median PSS score before the intervention in the group that received group psychotherapy plus yoga Vidya exercises intervention was 21.5, which was decreased to 18.9 after intervention. The statistical analysis did not reveal a significant difference in stress perception assessed by PSS in the two groups either before or after intervention Figure 6.

## 4. Discussion

Mental health professionals are susceptible to severe distress while caring for people with mental illness and this could decrease quality of life. Mental health professionals have to learn how to cope with distress, improve their coping abilities and adaptation to stress, sustain a good level of quality of life and a general psychological well-being. Our study showed that weekly group psychotherapy combined with yoga practice over a 12-week period did make a stress decrease not statistically significant, in opposition to the findings of Lin *et al.* (2015). In their study, Lin *et al.* demonstrated that yoga signiﬁcantly reduced work-related stress of mental health professionals. Their ﬁndings were consistent with the study of Wolever *et al.* (2012), showing that a psychological program, significantly improved perceived stress perception. Wolever *et al.* found that a 12-week (12 h) of psychological intervention program in a large American insurance company (*n* = 239) produced significant improvements in perceived stress differently from a control group that received no intervention. All these studies suggest that psychotherapy interventions are helpful in reducing stress perception, improve quality of life and general psychological well-being in mental health professionals. It is not clear why group psychotherapy plus yoga exercise made work-related stress far lower but did not enhance stress adaptation in mental health professionals. This may depend on group psychotherapy plus yoga practice, which can balance physiological and autonomic functions of distress, but not provide the skills for distress adaptation and general psychological well-being. The results of another trial (Hartfiel *et al.*, 2011) show that a six-week program of yoga had substantial positive effects of yoga for the improvement of the emotional well-being and resilience to stress among a randomized group of adults employed at a British university. The increase in scores for the yoga group during the six-week study period was substantially and significantly greater than that one of the control group. This indicates clear support for their hypothesis that a short six-week program of Dru Yoga can be effective for enhancing emotional well-being and resilience to stress in a workplace environment. These results are generally consistent with another randomized (but not controlled) published study (Granath *et al.*, 2006), which also found that psychotherapy plus yoga can be effective for improving psychological well-being. The statistically significant changes in perceived stress and psychological well-being scores for the yoga group, relative to the control group at the end of the 12-week period, give clear support for our hypothesis that a 12-week program of group psychotherapy plus yoga can be effective for reducing stress and for enhancing psychological well-being. Although our study and that one of Granath *et al.* (2006) found group psychotherapy plus yoga to be effective for improving general psychological well-being, however, these studies used different assessment tools and measured different populations. In their study of 33 employees in a large Swedish company, Granath *et al.* (2006) used the PSS and Quality of Life Index (QOLI) and found that 10 weekly sessions of cognitive behavioral therapy and yoga had a significant effect in reducing perceived stress, stress behavior and exhaustion but not in the Quality of Life Index, in opposition to our results. In fact, in our study the median QoL-VAS score revealed a significant difference between the quality of life assessed by Euro-QoL VAS between active group vs control group, while the median PSS score did not reveal a significant difference between the stress perception assessed by PSS in the two groups either before or after intervention. The limited data available do not permit a definitive conclusion to be drawn because several factors limit the conclusions that can be drawn from our study. First, we placed no restrictions on the activities of the control group during the intervention period. Similarly, we cannot exclude the possibility that the (unregulated) activities of the control group during the study period influenced their end-program scores. Additional limitations include our modest number of participants (*N* = 31) in comparison with other studies and the fact that we did not evaluate the long-term effects of group psychotherapy plus yoga on psychological well-being, quality of life and resilience to stress.

Future studies should collect data on absence rates and include an economic evaluation to determine the cost-effectiveness group psychotherapy plus yoga.

## Figures and Tables

**Table I:** Composition of the sample: sex

**Group**	Men	Women	Total
**Intervention**	19	12	31
**Control**	16	12	28

**Table II:** Composition of the sample: age

**Group**	Mean	St. Dev
**Intervention**	49.3	10.5
**Control**	48.9	10.1
